# Predicting the Solubility of Pharmaceutical Cocrystals in Solvent/Anti-Solvent Mixtures

**DOI:** 10.3390/molecules21050593

**Published:** 2016-05-07

**Authors:** Linda Lange, Stefan Heisel, Gabriele Sadowski

**Affiliations:** Department of Chemical and Biochemical Engineering, Laboratory of Thermodynamics, TU Dortmund University, Emil-Figge-Str. 70, D-44227 Dortmund, Germany; Linda.Lange@bci.tu-dortmund.de (L.L.); Stefan.Heisel@bci.tu-dortmund.de (S.H.)

**Keywords:** cocrystal, crystal engineering, thermodynamics, solubility, phase diagram, solvent mixtures

## Abstract

In this work, the solubilities of pharmaceutical cocrystals in solvent/anti-solvent systems were predicted using PC-SAFT in order to increase the efficiency of cocrystal formation processes. Modeling results and experimental data were compared for the cocrystal system nicotinamide/succinic acid (2:1) in the solvent/anti-solvent mixtures ethanol/water, ethanol/acetonitrile and ethanol/ethyl acetate at 298.15 K and in the ethanol/ethyl acetate mixture also at 310.15 K. The solubility of the investigated cocrystal slightly increased when adding small amounts of anti-solvent to the solvent, but drastically decreased for high anti-solvent amounts. Furthermore, the solubilities of nicotinamide, succinic acid and the cocrystal in the considered solvent/anti-solvent mixtures showed strong deviations from ideal-solution behavior. However, by accounting for the thermodynamic non-ideality of the components, PC-SAFT is able to predict the solubilities in all above-mentioned solvent/anti-solvent systems in good agreement with the experimental data.

## 1. Introduction

Cocrystals (CCs) are formulations of increasing interest to the pharmaceutical industry, as they can potentially improve the solubility and dissolution behavior, as well as the physical stability of active pharmaceutical ingredients (APIs) compared to their pure state [1,2,3,4,5,6]. CCs consist of an API and at least one coformer (CF) in a stoichiometric ratio. Because of the weak non-covalent interactions between the API and CF in the CC, it dissociates into its components upon dissolution. 

In general, CC formation is performed by crystallization from solution, for example by cooling or by the addition of anti-solvents [7]. Anti-solvent crystallization is a favored technique, as it offers an extended range of solvent polarity compared to single solvents [8]. This in turn provides the possibility to control crystal properties, such as purity [9], crystal size [10,11], morphology [10], crystal size distribution [12], agglomeration [13,14] and polymorphism [15,16,17]. Other studies investigated the influence of solvent/anti-solvent systems on crystal nucleation, the size of the metastable zone and crystal growth during crystallization processes [11,16,18,19,20]. Moreover, Rager and Hilfiger [21] demonstrated that mixtures of solvents or solvent/anti-solvent favor CC formation by suppressing solvate formation. However, anti-solvent crystallization is only a reasonable alternative to cooling crystallization when the solubility of the desired CC is drastically decreased in the anti-solvent/solvent mixture compared to the pure solvent [22].

As shown in previous studies [23,24,25,26], effective CC formation by crystallization from solution requires the knowledge of the thermodynamic phase diagram for a given system of API, CF, solvent and, if appropriate, anti-solvent. The reliable and accurate experimental investigation of these diagrams is time consuming and expensive [25,27,28,29,30]. Therefore, several approaches were developed to determine the concentration range in an API/CF/solvent system in which stable CCs are formed [3,23,30,31,32,33,34].

In most of these approaches, the CC formation is modeled as a chemical reaction of the liquid API and the liquid CF, resulting in the solid CC. The solubility of the CC can be obtained from the equilibrium constant of this reaction, the so-called solubility product [23,26,35]:

(1) $$K_{s}=\underset{i}{\prod }{a_{i}}^{\nu_{i}}={\left(x_{API}\;\gamma_{API}\right)}^{\nu_{API}}\cdot {\left(x_{CF}\;\gamma_{CF}\right)}^{\nu_{CF}}$$

In Equation (1), $x_{i\;}$ and $\gamma_{i}$ are the solubility mole fractions and corresponding activity coefficients of API and CF, respectively. $a_{i}$ is the activity of these components, while the activity of the solid CC is equal to one. $\nu_{API}$ and $\nu_{CF}$ correspond to the stoichiometry of API and CF in the CC. The solubility product $K_{s}$ does not depend on the solvent or solvent/anti-solvent mixture. Further, it does not depend on the concentration, but only varies with temperature. Thus, it can be calculated based on only one CC solubility data point, regardless of solvent, solvent/anti-solvent mixture or concentration.

However, in many studies, the solubility product is calculated neglecting the API and CF activity coefficients. The resulting $K_{s}^{\;ideal}$ is shown in Equation (2).

(2) $$K_{s}^{\;ideal}=\frac{K_{s}}{\prod_{i}{\left(\gamma_{i}\right)}^{\nu_{i}}}=\frac{\prod_{i}{\left(a_{i}\right)}^{\nu_{i}}}{\prod_{i}{\left(\gamma_{i}\right)}^{\nu_{i}}}=\underset{i}{\prod }{x_{i}}^{\nu_{i}}={\left(x_{API}\right)}^{\nu_{API}}\;{\left(x_{CF}\right)}^{\nu_{CF}}$$

In the literature, there exist only a few studies investigating the CC phase behavior in solvent (or solvent/anti-solvent) mixtures [21,36,37,38,39,40,41]. Solely, Sheikh *et al.* [36], Wang *et al.* [39] and Lee *et al.* [37] estimated CC solubility in solvent/anti-solvent mixtures using a solubility product. However, all of them calculated $K_{s}^{\;ideal}$ via Equation (2), neglecting the activity coefficients of the components forming the CC. Since this $K_{s}^{\;ideal}$ is only valid for one particular CC solubility point, as well as for one particular solvent/anti-solvent mixture, it is not applicable for predictions in different solvents or solvent/anti-solvent mixtures. Sun *et al.* [40] and Nishimaru *et al.* [41] emphasized the advantage of symmetrical ternary phase diagrams in view of a simple process design for CC formation via anti-solvent crystallization. This phenomenon, characterized by similar solubilities of API and CF in the anti-solvent/solvent mixture (congruent dissolution [24]), is not necessary, but beneficial for successful cocrystal formation [23].

In contrast to the above-mentioned approaches, this work considers the thermodynamic non-ideality of all components in API/CF/solvent/anti-solvent systems in order to identify feasible solvent/anti-solvent mixtures for efficient CC formation. For this purpose, the activity coefficients in Equation (1) were calculated using the perturbed-chain statistical associating fluid theory (PC-SAFT). PC-SAFT was already used earlier to model and predict the solubility in binary and ternary systems [42,43,44,45,46] and also systems exhibiting CC formation [23,34,47]. In a prior study, CC solubilities in different solvents and at different temperatures could be predicted for various CC systems in high accordance with experimental data [23]. Moreover, Ruether and Sadowski [35], as well as Fuchs *et al.* [48] presented PC-SAFT as a powerful tool for reliable solubility predictions of APIs and API-like components in solvent mixtures. 

In this work, the CC composed of nicotinamide (NA) and succinic acid (SA) was considered in the solvent/anti-solvent systems ethanol/water, ethanol/acetonitrile, as well as ethanol/ethyl acetate. According to the Food and Drug Administration, these solvents/anti-solvents may be present in APIs (Class 2 and 3 solvents) [49]. Ethanol, water and ethyl acetate are even regarded as solvents of low toxic potential, so-called Class 3 solvents [49]. 

## 2. Theory

### 2.1. Solubility Calculations

The solubility of API or CF can be calculated accounting for a thermodynamic equilibrium between a pure solid phase and the liquid (melt or solution), as follows [50]:

(3) $$x_{i}^{L}=\frac{1}{\gamma_{i}^{L}}exp\left[-\frac{\Delta h_{0i}^{SL}}{RT}\left(1-\frac{T}{T_{0i}^{SL}}\right)-\frac{\Delta c_{P,0i}^{SL}}{R}\left(\frac{T_{0i}^{SL}}{T}-1-\text{ln}\frac{T_{0i}^{SL}}{T}\right)\right]$$

In Equation (3), $x_{i}^{L}$ is the solubility (mole fraction) of component i (API or CF) in the liquid phase. *T* is the temperature of the system, whereas R is the ideal gas constant. $T_{0i}^{SL}$ is the melting temperature, indicating the temperature at which the (pure) component i (API or CF) changes state from solid to liquid. $\Delta h_{0i}^{SL}$ is the heat of fusion of component i (API or CF), assumed to be a constant over the considered temperature range. $\Delta c_{P,0i}^{SL}$ describes the difference in the solid and liquid heat capacities of component i at its melting point. $\gamma_{i}^{L}$ represents the activity coefficient of API or CF in the liquid phase that depends on temperature and on the concentrations of all components in the liquid mixture. All activity coefficients considered in this work were calculated by PC-SAFT.

The thermodynamic equilibrium condition in Equation (3) was derived based on the equality of the chemical potentials of one solute in the solid and in the liquid phase. However, the CC decomposes into its components, API and CF, upon dissolution. Consequently, the CC does not exist in the liquid phase, but is totally dissolved into API and CF. Thus, CC solubility cannot be modeled using Equation (3), but is described via the solubility product $K_{s}$ (Equation (1)).

According to Equation (4), the temperature dependence of the solubility product $K_{s}$ can be estimated applying the Gibbs–Helmholtz equation.

(4) $$\ln K_{s}=lnK_{s}^{ref}+\frac{\Delta h_{s}^{ref}}{R}\left(\frac{1}{T^{ref}}-\frac{1}{T}\right)$$

$K_{s}^{ref}$ represents a reference solubility product at a reference temperature $T^{ref}$. The reference enthalpy $\Delta h_{s}^{ref}$ refers to the enthalpy of fusion of the complex at the reference temperature $T^{ref}$. $\Delta h_{s}^{ref}$ is assumed to be constant over a relatively small temperature range.

### 2.2. PC-SAFT

All required activity coefficients$\;\gamma_{i}$ in this work (Equations (1) and (3)) were calculated using PC-SAFT. A detailed derivation of PC-SAFT is given in the literature [42,43]. It is based on a perturbation theory using a hard-chain reference system. The residual Helmholtz energy $a^{residual}$ equals the sum of different contributions that are considered independently, namely that accounting for repulsive interactions (hard chain), for attraction (dispersion), as well as for hydrogen-bonding interactions (association):

(5) $$a^{residual}=a^{hard\;chain}+a^{dispersion}+a^{association}$$

In PC-SAFT, three pure-component parameters are used to characterize a non-associating molecule: the number of segments $m_{i}^{seg}$, the segment diameter $\sigma_{i}$ and the dispersion-energy parameter $u_{i}/k_{B}$ [42]. As most of the components investigated in this work are able to associate, two additional parameters were considered: the association-energy parameter$\;\varepsilon^{AiBi}/k_{B}$ and the association-volume parameter $\kappa^{AiBi}$ [43].

Applying Berthelot–Lorentz combining rules, the segment diameter $\sigma_{ij}$ and the dispersion-energy parameter $u_{ij}$ in mixtures of substances i and j were estimated via Equations (6) and (7) [51]. An additional binary interaction parameter $k_{ij}$ was required correcting the cross-dispersion energy between the substances i and j.

(6) $$\sigma_{ij}=\frac{1}{2}\left(\sigma_{i}+\sigma_{j}\right)$$

(7) $$u_{ij}=\left(1-k_{ij}\right)\sqrt{u_{i}u_{j}}$$

The cross-associating interactions between two associating components i and j were modeled using combining rules derived by Wolbach and Sandler [52] (Equations (8) and (9)).

(8) $$\varepsilon^{AiBj}=\frac{1}{2}\left(\varepsilon^{AiBi}+\varepsilon^{AjBj}\right)$$

(9) $$\kappa^{AiBj}=\sqrt{\kappa^{AiBi}\kappa^{AjBj}}{\left(\frac{\sqrt{\sigma_{i}\sigma_{j}}}{\left(1/2\right)\left(\sigma_{i}+\sigma_{j}\right)}\right)}^{3}$$

In this work, also mixtures of a polar, but non-self-associating component and a self-associating component were investigated. As described by Kleiner and Sadowski [53], in these cases, the induced association of the non-self-associating component was assumed. For that purpose, the association-energy parameter $\varepsilon^{AiBi}$ of the non-self-associating component was set to zero, and its association-volume parameter $\kappa^{AiBi}$ was set to 0.01. The number of association sites for associating and induced associating components was chosen according to the association schemes postulated by Huang and Radosz [54].

## 3. Materials and Experimental Methods

### 3.1. Materials

NA (purity >99%) was purchased as crystalline powder from Sigma-Aldrich (Hamburg, Germany), and SA, also used as crystalline powder (99.8%), was obtained from VWR Chemicals (Darmstadt, Germany). Acetonitrile (>99.9%) and ethyl acetate (≥99.5%) were also purchased from VWR Chemicals, whereas ethanol (≥99.9%) was purchased from Merck KGaA (Darmstadt, Germany). Potassium dihydrogen phosphate (≥99%) was obtained from Sigma-Aldrich, dipotassium phosphate (99.8%) from VWR Chemicals and sodium hydroxide as pellets (≥98%) from Bernd Kraft GmbH (Duisburg, Germany). All substances were used without further purification as obtained from the manufactures. Water was filtered, deionized and distilled with a Millipore purification system.

### 3.2. Solubility in Solvents, Anti-Solvents and Solvent/Anti-Solvent Mixtures

Solubility measurements of NA, SA and mixtures of NA/SA were performed in ethanol, ethyl acetate, acetonitrile, as well as in ethanol/water, ethanol/acetonitrile and ethanol/ethyl acetate mixtures. The corresponding solubility experiments executed in this work are listed in Table 1.

The solubility measurements were performed by preparation of a saturated solution of the pure solute (NA or SA) or solute mixtures (NA/SA) in the (anti-)solvent or solvent/anti-solvent mixture. The solution was mixed in a glass vessel (50 mL) using a magnetic stirrer to ensure sample equilibration. The temperature of the solution was adjusted by a heating jacket and measured by a PT100 element (accuracy of ±0.1 K). All samples were equilibrated for at least 48 h. After equilibration, samples were taken from the liquid phase and filtered (pore size 0.45 µm). To avoid nucleation during sampling, the syringes (10 mL) and needles were preheated.

The concentrations of pure NA or SA in saturated solutions of ethanol, ethyl acetate and the ethanol/ethyl acetate mixtures were determined gravimetrically. 

The concentrations of NA in saturated solutions of the remaining solvents and solvent/anti-solvent mixtures—namely, acetonitrile, ethanol/water mixture (0.5/0.5 *w*/*w*) and ethanol/acetonitrile mixture (0.67/0.33 *w*/*w*)—were measured via UV-VIS spectrometry (Eppendorf BioSpectrometer^®^, Hamburg, Germany) at 260 nm. The calibration curve, prepared prior to the experiments, resulted in a coefficient of calibration of 0.99995.

The solubilities of SA in solvents and solvent/anti-solvent mixtures containing acetonitrile, ethanol, water or mixtures of these were determined by high-performance liquid chromatography (HPLC) from Agilent Technologies (1200 Series) and a reversed-phase column (Eclipse XDB-C18, 5 µm, 4.6 mm × 150 mm), again measuring concentrations via UV-VIS spectrometry, but here at 210 nm. The HPLC worked with a mobile phase consisting of 50 mM phosphate buffer at pH 7 and acetonitrile (gradient from 100/0 *v*/*v* to 60/40 *v*/*v*).

The concentrations of NA and SA in mixed solutions of both were determined as described above. The concentration of NA was analyzed via UV-VIS spectrometry at 260 nm, whereas that of SA was determined by HPLC and UV-VIS spectrometry at 210 nm. Samples containing ethyl acetate as solvents were dissolved in water prior to photometric analysis. For that purpose, ethyl acetate was evaporated by a vacuum concentrator (IR Micro-Cenvac NB-503CIR, N-BIOTEK Co., Ltd., Gyeonggi-do, Korea) before the remaining NA/SA mixture was dissolved in water.

Every gravimetric and photometric analysis was executed three times, and the average value is reported. Additionally, the solid phase of each saturated solution was analyzed by X-ray diffraction (XRD, Miniflex, Rigaku, Japan). The experimentally-determined solubilities, their standard deviations and the corresponding solid phases are reported in the Supplementary Material.

### 3.3. Melting Temperatures of NA and SA

Besides X-ray diffraction, the solid phases of NA and SA were characterized by their temperature $T_{0i}^{SL}$ measured using a modulated differential scanning calorimeter (DSC). The apparatus (Q2000, TA Instruments GmbH, Eschborn, Germany) was calibrated against the melting temperature and melting enthalpy of pure indium. Samples of NA (or SA) of around 3.5 mg were heated in hermetically-sealed aluminum pans (TA Instruments GmbH) with a modulated heating rate of 2 K·min^−1^ to approximately 20 K above the expected melting temperature. During the measurement, the apparatus was flushed by nitrogen with a flow rate of 40 mL·min^−1^ to ensure an inert atmosphere. The samples were analyzed using TA Universal Analysis 2000 Software (TA Instruments GmbH). It could be ensured that every sample of NA corresponds to the polymorphic form I. Further, all samples of SA showed melting temperatures identical to that of the supplied SA, so that esterification reactions with ethanol could be excluded.

## 4. Results and Discussion

### 4.1. Solubility of NA and SA in Pure (Anti-)Solvents

The solubilities of NA and SA in pure (anti-)solvents were correlated using PC-SAFT for calculating the solute activity coefficients $\gamma_{i}^{L}$ (Equation (3)). The application of PC-SAFT requires the pure-component parameters of NA, SA and of all considered (anti-)solvents, namely acetonitrile, ethanol, ethyl acetate and water. 

Additionally, binary interaction parameters $k_{ij}$ between the NA (or SA) and the respective (anti-)solvents were considered. As shown in Equation (10), $k_{ij}$ was assumed to linearly depend on temperature:

(10) $$k_{ij}\left(T\right)=k_{ij,slope}T+k_{ij,int}$$

**Figure 1:**
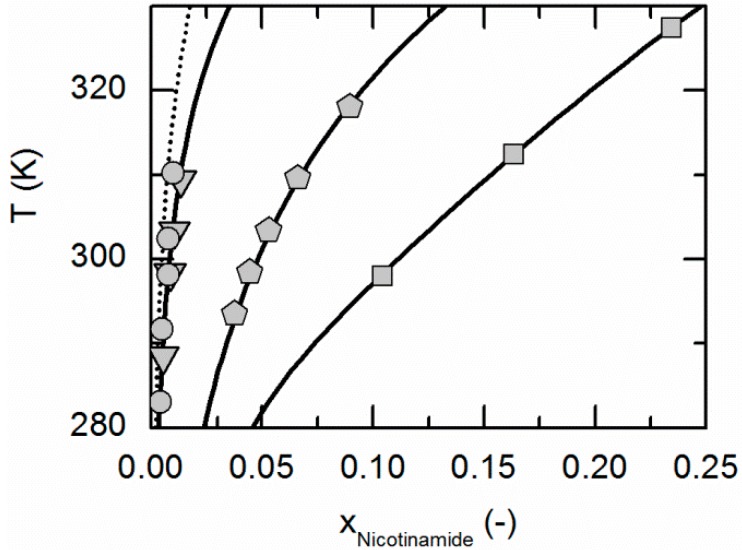
Solubility of nicotinamide in ethyl acetate (circles, dotted line), acetonitrile (triangles), ethanol (pentagons) and water [59] (squares). All lines correspond to the PC-SAFT correlations; symbols represent the experimental data points.

The melting properties of NA and SA required for the solubility calculations (Equation (3)) were obtained from the literature (Table 2). The same applies for the pure-component parameters used in this work (Table 3). The non-zero binary interaction parameters used in this work are listed in Table 4. In this study, binary interaction parameters between NA (or SA) and ethyl acetate were fitted to solubility data in the same solvent (Figure 1) as described by Ruether and Sadowski [35]. They are also included in Table 4.

**Figure 2:**
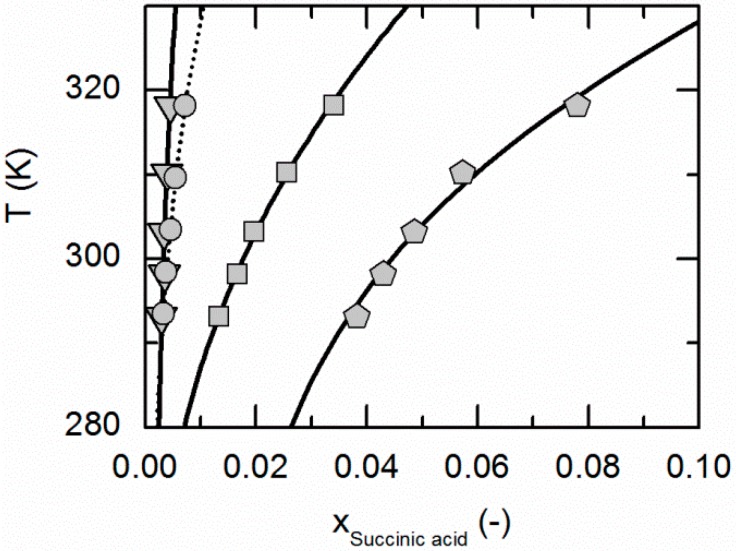
Solubility of succinic acid in ethyl acetate (circles, dotted line), acetonitrile [26] (triangles), ethanol [26] (pentagons) and water [26] (squares). Lines correspond to the PC-SAFT correlations; symbols represent the experimental data points.

Figure 1 compares the correlated modeling results for the solubility of NA in all solvents considered in this work, namely ethyl acetate, acetonitrile, ethanol and water. The solubility is highest in water, followed by ethanol, acetonitrile and ethyl acetate. Figure 2 shows the experimentally-obtained and correlated solubility data of SA in various solvents. In contrast to NA, the solubility is highest in ethanol, followed by water, ethyl acetate and acetonitrile.

To evaluate the accuracy of the solubility calculations, the average relative deviation (ARD) between the calculated and experimentally-determined solubility data of NA and SA was estimated:

(11) $$ARD=100\;\frac{1}{n_{exp}}\;\overset{n_{exp}}{\underset{i=1}{\sum }}\left|\frac{x_{calc,i}-x_{exp,i}}{x_{exp,i}}\right|$$

$x_{exp,i}$ is the experimental solubility; $x_{calc,i}$ is the corresponding calculated value; and $n_{exp}$ is the number of experimental solubility data points. The resulting ARDs of NA and SA between the calculated and experimentally-determined solubilities in pure solvents are summarized in Table 4. 

The ARDs are lower than 2.65% for the SA solubilities in all considered solvents. The same applies for the solubility of NA in ethanol and water, whereas the ARDs in acetonitrile and ethyl acetate are a bit higher (9.34% and 17.5%, Table 4). However, from Figure 1 and Figure 2, it becomes obvious that the solubility of both, NA and SA, is much lower in acetonitrile and ethyl acetate than in ethanol and water. This in turn probably causes the relatively high ARDs for the solubility calculations of NA in acetonitrile and ethyl acetate (compare Table 4). However, the almost quantitative agreement of remaining solubility correlations with experimental data demonstrates that PC-SAFT allows for an accurate correlation of the solubility data.

### 4.2. Solubility of NA and SA in Solvent/Anti-Solvent Mixtures at 298.15 K

In a second step, the solubilities of NA and SA in solvent mixtures were calculated (using again Equation (3), together with the pure-component and binary interaction parameters from Table 2 and Table 3). The symbols in the following figures, representing the solubility of NA in a pure solvent, are equivalent to the symbols in Figure 1. The same applies for SA (shown in Figure 2). The meaning of all symbols used in this work is listed in Table 5.

**Figure 3:**
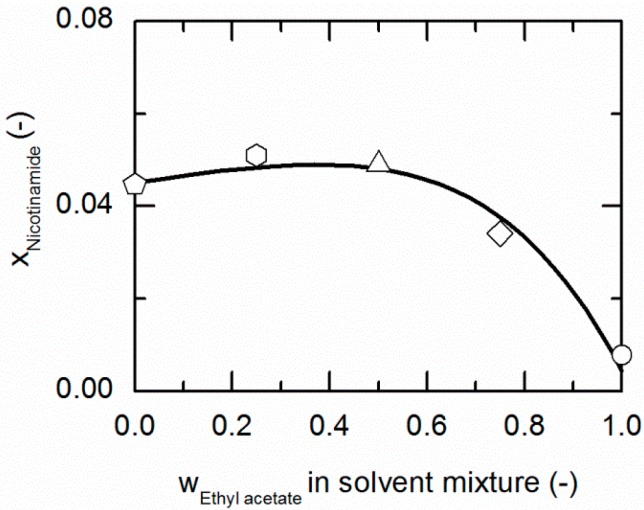
Solubility of nicotinamide (NA) in ethanol/ethyl acetate mixtures at 298.15 K. The line correspond to the PC-SAFT correlation; symbols represent the experimental data points for NA in ethanol (pentagon), in the ethanol/ethyl acetate (solute-free) mixtures 0.75/0.25 *w*/*w* (hexagon), 0.5/0.5 *w*/*w* (up-pointing triangle), 0.25/0.75 *w*/*w* (diamond) and in ethyl acetate (circle).

**Figure 4:**
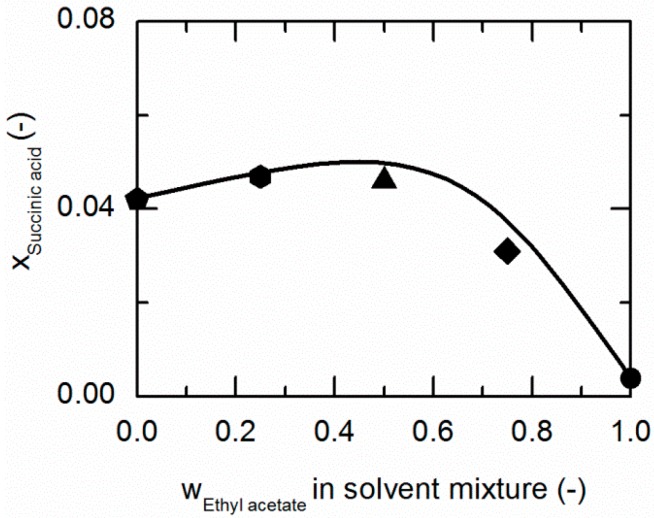
Solubility of succinic acid (SA) in ethanol/ethyl acetate mixtures at 298.15 K. The line correspond to the PC-SAFT correlation; symbols represent the experimental data points for SA in ethanol (pentagon), in the ethanol/ethyl acetate (solute-free) mixtures 0.75/0.25 *w*/*w* (hexagon), 0.5/0.5 *w*/*w* (up-pointing triangle), 0.25/0.75 *w*/*w* (diamond), and in ethyl acetate (circle).

Figure 3 and Figure 4 represent the solubilities of NA and SA in solvent/anti-solvent mixtures shown for ethanol/ethyl acetate mixtures as an example. Figure 3 compares the modeling results for the solubilities of NA at 298.15 K with experimental data. The data point on the left axis refers to the NA solubility in pure ethanol, whereas that one on the right axis corresponds to the solubility in pure ethyl acetate. Over the whole range of ethanol/ethyl acetate mixtures, the solubility of NA shows a positive deviation from a straight line between the solubilities in pure ethanol and ethyl acetate. By the addition of ethyl acetate to ethanol up to a mass fraction of 0.5/0.5 in the (solute-free) solvent mixture, the solubility of NA increases slightly until it reaches a maximum. For higher mass fractions of ethyl acetate, the solubility drastically decreases again. 

As demonstrated in Figure 4, SA shows a similar behavior in ethanol/ethyl acetate mixtures. Again, an addition of ethyl acetate to ethanol causes a slight increase in the solubility of SA up to mass fractions of 0.5/0.5 in the (solute-free) solvent mixture before it drastically decreases. 

The solubilities of NA and SA are very similar in ethanol/ethyl acetate mixtures. As postulated by Chiarella *et al.* [24], very similar solubilities of API and CF are likely to cause the so-called congruent dissolution behavior in ternary API/CF/solvent systems, which is in turn quite advantageous for the formation of the pharmaceutical CC investigated within this work, consisting of NA and SA (2:1).

Table 6 summarizes the ARDs of NA and SA in solvent/anti-solvent mixtures. The modeling accuracy for NA (ARDs < 5.86) is higher than for SA (ARDs < 31.76). However, the ARD of 31.76% for the SA solubility in ethanol/water mixtures is the highest estimated in this work. As already shown earlier, modeling of ethanol/water mixtures is particularly difficult due to the strong non-ideal behavior of this solvent/anti-solvent mixture [63].

### 4.3. Solubility of NA and SA in Solvent/Anti-Solvent Mixtures at 310.15 K

In this section, the solubilities of NA and SA in solvent mixtures are predicted for 310.15 K. These predictions were performed again for the solvent/anti-solvent system ethanol/ethyl acetate. 

**Figure 5:**
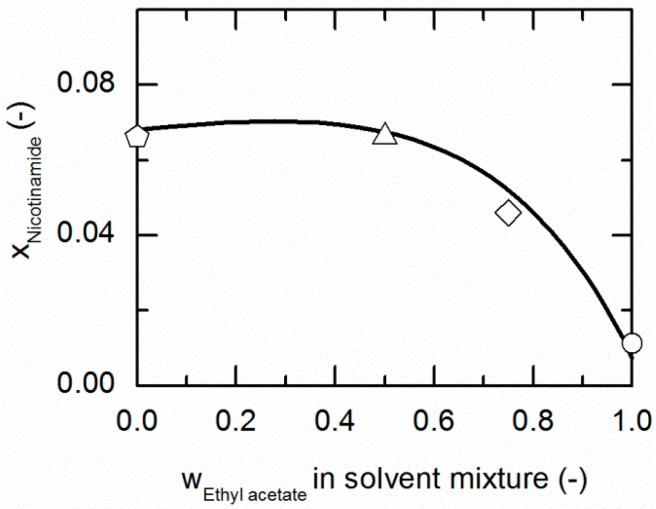
Solubility of nicotinamide (NA) in ethanol/ethyl acetate mixtures at 310.15 K. The line correspond to the PC-SAFT prediction; symbols represent the experimental data points for NA in ethanol (pentagon), in the ethanol/ethyl acetate (solute-free) mixtures 0.5/0.5 *w*/*w* (up-pointing triangle), 0.25/0.75 *w*/*w* (diamond) and in ethyl acetate (circle).

Figure 5 represents the solubilities of NA in ethanol/ethyl acetate mixtures at 310.15 K, which are about one and a half-times bigger than the solubilities at 298.15 K (shown in Figure 3). The qualitative course of the solubilities as a function of the considered ethanol/ethyl acetate mixtures remains the same as for 298.15 K. As to be seen from Figure 3, the solubilities in the solvent mixture can be predicted in good agreement with the experimental data (compare Table 6).

**Figure 6:**
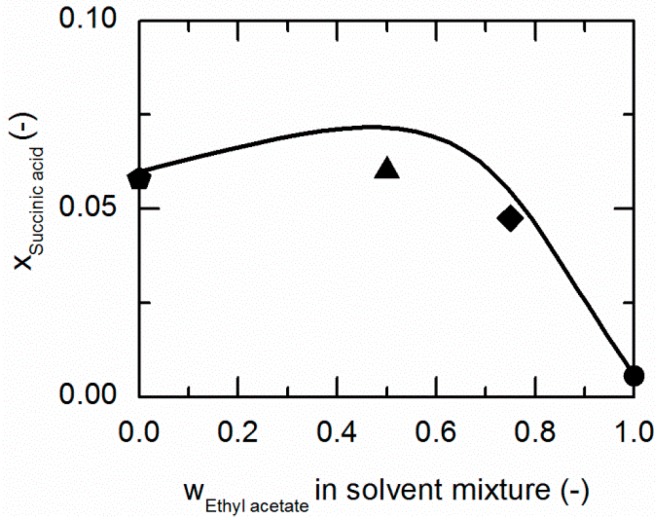
Solubility of succinic acid (SA) in ethanol/ethyl acetate mixtures at 310.15 K. The line correspond to the PC-SAFT prediction; symbols represent the experimental data points for SA in ethanol (pentagon), in the ethanol/ethyl acetate (solute-free) mixtures 0.5/0.5 *w*/*w* (up-pointing triangle), 0.25/0.75 *w*/*w* (diamond) and in ethyl acetate (circle).

Figure 6 compares the modeled and experimentally-determined solubility data of SA in ethanol/ethyl acetate mixtures at 310.15 K. Compared to 298.15 K (illustrated in Figure 4), the solubilities in all considered ethanol/ethyl acetate mixtures increased. The predicted solubility line is in qualitative agreement with the experimental data (ARDs < 11.11; Table 6).

### 4.4. Solubility Predictions in the NA/SA/Solvent/Anti-Solvent System at 298.15 K

The solubility of NA in the presence of SA and *vice versa* was again calculated using Equation (3). The binary interaction parameter between NA and SA was set to zero. 

The solubility of the CC, consisting of NA and SA in a 2:1 stoichiometry, was calculated using Equation (1). The CC solubility product $K_{s}$ was determined on the basis of only one CC solubility data point in water taken from a former publication [26]. The resulting $K_{s}$ is listed in Table 7.

**Figure 7:**
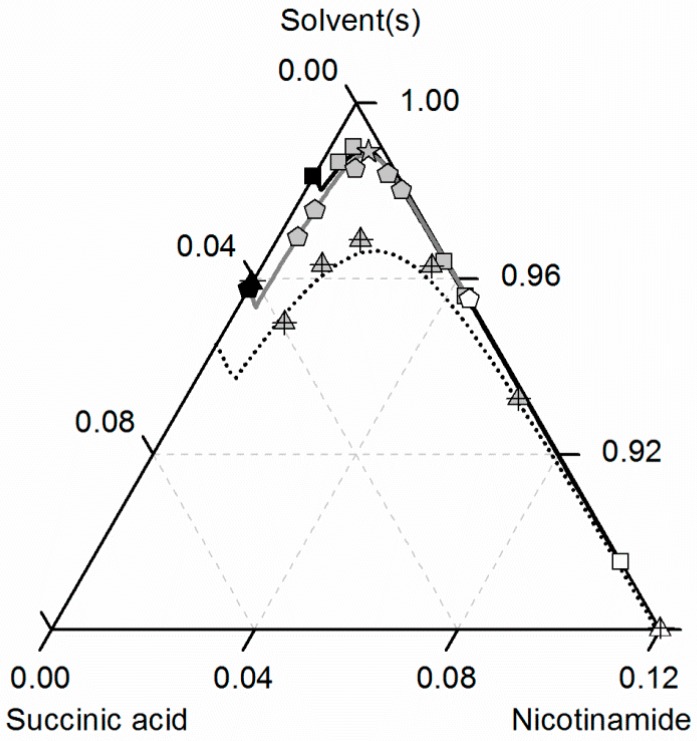
Solubilities of nicotinamide (NA) and succinic acid (SA) and the corresponding 2:1 cocrystal (CC) in ethanol (pentagons, gray solid lines), water (squares, black solid lines) and an ethanol/water mixture 0.5/0.5 *w*/*w* (crossed triangles, black dotted lines) at 298.15 K with shortened axes in mole fractions. Lines correspond to the PC-SAFT calculations; symbols refer to the experimental data points of SA (black), the CC (gray) and NA (white). The star is the CC solubility point used for the solubility product $K_{s}$. The CC lines in ethanol and the ethanol/water mixture were fully predicted.

Figure 7 depicts the solubilities of NA, SA and the corresponding cocrystal (2:1) in water, ethanol and an ethanol/water mixture 0.5/0.5 *w*/*w*. The solubility of NA is highest in the ethanol/water mixture 0.5/0.5 *w*/*w*, followed by pure water and pure ethanol. In the case of SA, the highest solubility can be observed in ethanol, followed by that in the ethanol/water mixture 0.5/0.5 *w*/*w* and, finally, water. This in turn means that in the case of NA, ethanol is the anti-solvent of the ethanol/water system, whereas water is the anti-solvent in the case of SA. This results in a highly asymmetrical ternary phase diagram. In contrast to NA and SA, the resulting CC is most soluble in the ethanol/water mixture 0.5/0.5 *w*/*w*, followed by ethanol and then water. Obviously, ethanol/water mixtures are not suitable for anti-solvent crystallization of the considered CC, as its solubility is not decreased by the addition of ethanol or water, but even significantly increased. Further, it is impossible to estimate this CC solubility behavior only based on the solubilities NA and SA, as proposed by ter Horst *et al.* [32].

As illustrated in Figure 7, the solubility of SA slightly increases by adding small amounts of NA, whereas the CC solubility strongly decreases by adding NA. Thus, the two solubility lines of NA and the CC shown in Figure 7 meet at a eutectic point that is visible as a kink.

Here, $K_{s}$ of the CC (Table 7) determined from one reference solubility point in water and the PC-SAFT-predicted activity coefficients of NA and SA in ethanol (and the ethanol/water mixture) were used to predict the CC solubility (Figure 7). Although ethanol/water mixtures are known for being strongly non-ideal [63], the modeling results show an almost quantitative agreement with the experimental data (ARD 4.29%, Table 6).

**Figure 8:**
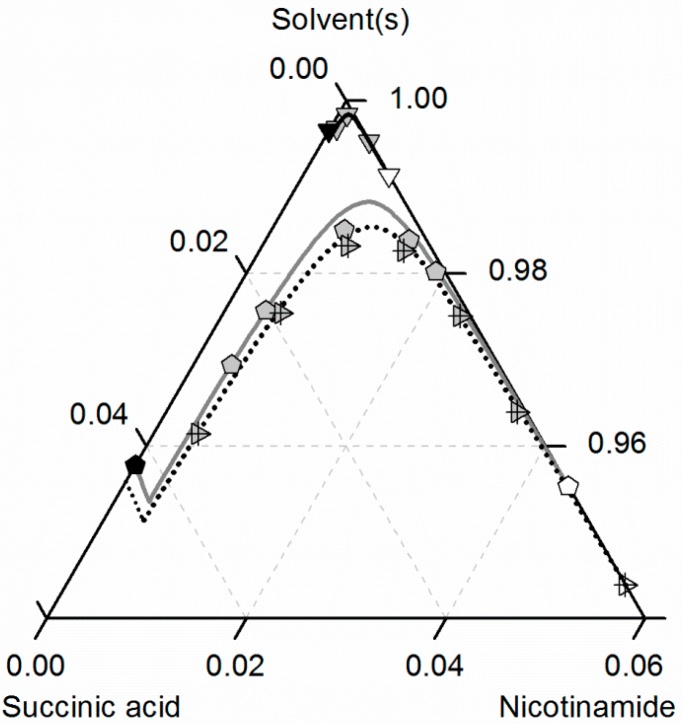
Solubilities of nicotinamide (NA) and succinic acid (SA) and the corresponding 2:1 cocrystal (CC) in ethanol (pentagons, gray solid lines), acetonitrile (triangle pointing downwards, black solid lines) and an ethanol/acetonitrile mixture 0.67/0.33 *w*/*w* (crossed triangle pointing to the right, black dotted lines) at 298.15 K with shortened axes in mole fractions. Lines correspond to the PC-SAFT predictions; symbols refer to the experimental data points of SA (black), the CC (gray) and NA (white).

Figure 8 compares the predicted and experimental data for the investigated CC in ethanol/acetonitrile mixtures. The solubilities in ethanol were adopted from Figure 7. In accordance with the solubilities of NA and SA, the CC solubility is slightly higher in the ethanol/acetonitrile mixture 0.67/0.33 *w*/*w* than in ethanol, resulting in an almost symmetrical phase diagram. In the case of SA, the experimental solubilities in the ethanol/acetonitrile mixture and in ethanol are so close that the symbols even overlap in Figure 7. In contrast, the solubilities of NA, SA and the corresponding CC are much lower in acetonitrile than in ethanol. Consequently, ethanol and acetonitrile behave significantly better as a solvent/anti-solvent system with regard to the investigated CC than ethanol and water (compare Figure 7). Again, the predicted solubilities are in high accordance with the experimental data points (ARD 5%, Table 6).

**Figure 9:**
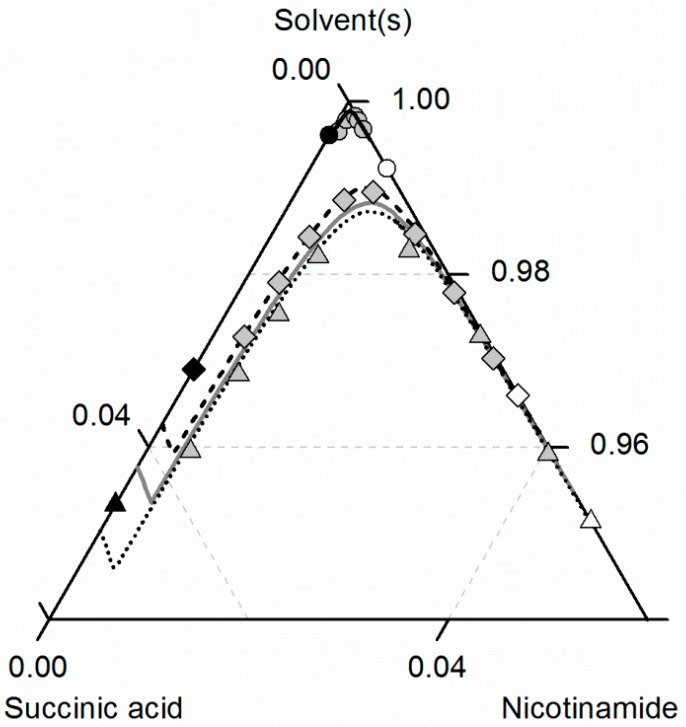
Solubilities of nicotinamide (NA) and succinic acid (SA) and the corresponding 2:1 cocrystal (CC) in ethanol (gray solid lines), ethyl acetate (circles, black solid lines) and ethanol-ethyl acetate mixtures 0.5/0.5 *w/w* (triangles, black dotted lines) and 0.25/0.75 *w*/*w* (diamonds, black dashed lines) at 298.15 K with shortened axes in mole fractions. Lines correspond to the PC-SAFT predictions; symbols refer to the experimental data points of SA (black), the CC (gray) and NA (white).

Finally, the solubilities of NA, SA and the corresponding CC in ethanol/ethyl acetate mixtures are shown in Figure 9. As can be seen in Figure 3 and Figure 4, the solubilities of NA and SA are quite similar for all investigated ethanol/ethyl acetate mixtures, resulting also in an almost symmetrical ternary phase diagram. For better visibility, only the correlated solubilities of NA, SA and the CC in ethanol are shown in Figure 9 instead of the experimentally-determined solubility data (illustrated in Figure 7 and Figure 8). The solubility data in the ethanol/ethyl acetate mixture 0.75/0.25 *w*/*w* are not illustrated at all, since the experimental, as well as the predicted solubilities overlap with that in the 0.5/0.5 *w*/*w* mixture. 

It becomes obvious that the solubilities of NA, SA and the CC in the ethanol/ethyl acetate mixture 0.5/0.5 *w*/*w* exceed those in pure ethanol. Only for higher concentrations of ethyl acetate in the solvent mixture (0.75 *w*/*w*), the CC solubility is lower than that in ethanol. For all solutes, the lowest solubilities were determined in ethyl acetate. Besides ethanol/acetonitrile, also the mixture of ethanol and ethyl acetate turns out to be an effective solvent/anti-solvent system for the investigated CC. Furthermore, the high symmetry of the ternary phase diagram for ethanol/ethyl acetate mixtures enables a simplified process design for CC formation. The ARDs of the predicted CC solubility lines lower than 3.85% for all considered ethanol/ethyl acetate mixtures verify the very good agreement between predictions and experimental data.

Table 8 represents the ARDs of CC solubilities in NA/SA/solvent/(anti-solvent) systems. The deviations of experimental and calculated data in Equation (11) refer to that of NA. The model accuracy for CC solubilities (ARDs <7.31%, Table 8) is even higher than for NA or SA (Table 3 and Table 4). The ARDs of CC solubilities in NA/SA/solvent/systems are lower than 2.58% for ethanol and water, but up to 6.68% in acetonitrile and ethyl acetate. ARDs for the CC solubility in solvent mixtures at 298.15 K are even lower than 5.00%. The highest ARD of 7.31% was obtained for the ethanol/ethyl acetate mixture at 310.15 K. Thus, the CC solubility could be predicted in all considered solvents, as well as in all solvent/anti-solvent mixtures in almost quantitative agreement with experimental data.

### 4.5. Solubility Predictions in the NA/SA/Solvent/Anti-Solvent System at 310.15 K

Finally, PC-SAFT was used to predict the CC solubility in solvent/anti-solvent mixtures at 310.15 K. The predictions were performed for solubilities in ethanol/ethyl acetate mixtures, using the previously-determined CC solubility product at 298.15 K and the CC reference melting enthalpy $\Delta h_{s}^{ref}$ taken from a former publication (compare Table 7) [26]. 

**Figure 10:**
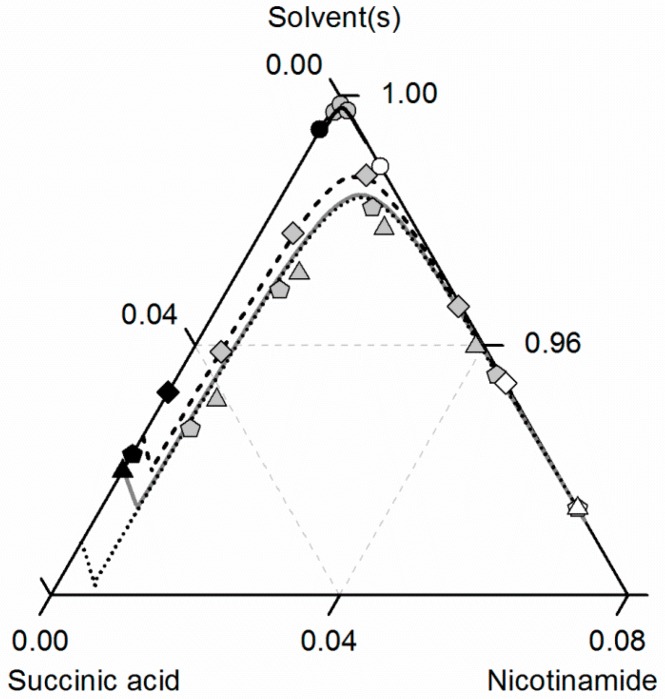
Solubilities of nicotinamide (NA) and succinic acid (SA) and the corresponding 2:1 cocrystal (CC) in ethanol (gray solid lines), ethyl acetate (circles, black solid lines), ethanol/ethyl acetate mixtures in (solute-free) solvent mixtures 0.5/0.5 *w*/*w* (triangles, black dotted lines) and 0.25/0.75 *w*/*w* (diamonds, black dashed lines) at 310.15 K with shortened axes in mole fractions. Lines correspond to the PC-SAFT predictions; symbols refer to the experimental data points of SA (black), the CC (gray) and NA (white).

Figure 10 compares predicted and experimental data for the investigated NA/SA/ethanol/ethyl acetate system at 310.15 K. The solubilities of NA, SA and of the CC increase for all investigated ethanol/ethyl acetate mixtures with temperature (see Figure 9), but the ternary phase diagram is less symmetric than at 298.15 K. In contrast to the data at 298.15 K, the CC solubility in ethanol at 310.15 K almost overlaps with the one in the ethanol/ethyl acetate mixture 0.5/0.5 *w*/*w*. Moreover, the CC solubility decrease when adding ethyl acetate to ethanol is more significant, as shown in Figure 9 for 298.15 K. Again, all modeled and predicted solubilities show very good agreement with the experimental data (Table 8).

## 5. Conclusions

This study represents a thermodynamically-correct description of the CC solubility behavior in solvent/anti-solvent mixtures using the example system NA and SA, which form a 2:1 CC in the investigated ethanol/water, ethanol/acetonitrile and ethanol/ethyl acetate mixtures.

The CC solubility in solvents or solvent/anti-solvent mixtures was modeled using the CC solubility product and accounting for thermodynamic non-idealities. The CC solubility product was obtained from only the CC solubility data point in water. Based on this, the CC solubility could be predicted in all considered solvents, as well as in all solvent/anti-solvent mixtures with high accordance to experimental data, allowing reliably determining the concentration range in which the CC is formed.

Further, employing the Gibbs–Helmholtz equation, the CC solubility could also be predicted in solvent/anti-solvent mixtures at another temperature. 

In summary, the proposed approach could be successfully applied for the identification of feasible solvent/anti-solvent mixtures for efficient CC formation.

## Figures and Tables

**Table 1:** Performed solubility measurements of nicotinamide (NA), succinic acid (SA) and mixtures of both in solvents or solvent/anti-solvent mixtures at specific temperatures.

Component(s)	Solvent or Solvent/Anti-Solvent Mixture	Temperature(s) (K)
NA	ethanol	293.15, 298.15, 303.15, 310.15, 318.15
NA	ethyl acetate	298.15, 310.15
NA	acetonitrile	288.15, 298.15, 303.15, 310.15
NA	ethanol/ethyl acetate mixtures (0.75/0.25 *w*/*w*, 0.5/0.5 *w*/*w*, and 0.25/0.75 *w*/*w*)	298.15
NA	ethanol/ethyl acetate mixtures (0.5/0.5 *w*/*w*, and 0.25/0.75 *w*/*w*)	310.15
NA	ethanol/water mixture (0.5/0.5 *w*/*w*)	298.15
NA	ethanol/acetonitrile mixture (0.67/0.33 *w*/*w*)	298.15
SA	ethyl acetate	293.15, 298.15, 303.15, 310.15, 318.15
SA	ethanol/ethyl acetate mixtures (0.75/0.25 *w*/*w*, 0.5/0.5 *w*/*w*, and 0.25/0.75 *w*/*w*)	298.15
SA	ethanol/ethyl acetate mixtures (0.5/0.5 *w*/*w*, and 0.25/0.75 *w*/*w*)	310.15
SA	ethanol/water mixture (0.5/0.5 *w*/*w*)	298.15
SA	ethanol/acetonitrile mixture (0.67/0.33 *w*/*w*)	298.15
NA, SA	ethyl acetate	298.15
NA, SA	acetonitrile	298.15
NA, SA	ethanol	298.15, 310.15
NA, SA	ethanol/ethyl acetate mixtures (0.75/0.25 *w*/*w*, 0.5/0.5 *w*/*w*, and 0.25/0.75 *w*/*w*)	298.15
NA, SA	ethanol/ethyl acetate mixtures (0.5/0.5 *w*/*w*, and 0.25/0.75 *w*/*w*)	310.15
NA, SA	ethanol/water mixture (0.5/0.5 *w*/*w*)	298.15
NA, SA	ethanol/acetonitrile mixture (0.67/0.33 *w*/*w*)	298.15

**Table 2:** Melting properties of nicotinamide (NA, form I) and succinic acid (SA).

Component	*T*_0*i*_*^SL^* (K)	∆*h*_0*i*_*^SL^* (kJ/mol)	∆*c_p_*_0*i*_*^SL^* (J/mol)	Ref.
NA(I)	401.15	28.0	78.12	[55,56]
SA	461.15	38.91	69.79	[57]

**Table 3:** PC-SAFT pure-component parameters for nicotinamide (NA) succinic acid (SA), and (anti-)solvents considered in this work.

Component	M (g/mol)	*m_i_* (−)	σ*_i_* (Å)	*u_i_* (K)	ε*^AiBi^* (K)	κ*^AiBi^* (−)	Association Scheme	Ref.
NA	122.12	4.6485	2.1775	176.69	2195.3	0.02	2/2	[26]
SA	118.09	4.3346	3.0546	477.44	1701.69	0.02	2/2	[26]
water	18.015	1.2047	^a^	353.94	2424.67	0.045	1/1	[48]
acetonitrile ^b^	41.052	2.3290	3.1898	311.31	0	0.01	-	[58]
ethanol	46.069	2.3827	3.1771	198.24	2653.4	0.03284	1/1	[43]
ethyl acetate ^b^	88.105	3.5375	3.3079	230.80	0	0.01	-	[42]

^a^ The expression σ = 2.7927 + 10.11 exp(−0.01775 × T) − 1.417 exp(−0.01146 × T) was used [48]. ^b^ Induced association in mixtures with associating components is considered.

**Table 4:** PC-SAFT binary interaction parameters ^a^ for binary sub-systems considered in this work and average relative deviations (ARDs) of calculated and experimental solubilities. All other binary parameters were set to zero.

	Binary Parameter		Temperature Range of the Experimental Data (K)	Ref for Parameters	Ref for Exp Data	ARD (%)
	*k_ij,slope_* (−)	*k_ij,int_* (−)				
API/solvent						
NA/water	9.46 × 10^−5^	−2.94 × 10^−2^	298–328	[26]	[59]	0.59
NA/acetonitrile	2.92 × 10^−4^	−8.46 × 10^−2^	288.47–309.4	[26]	this work	9.34
NA/ethanol	8.39 × 10^−5^	−2.10 × 10^−2^	293.51–318.05	[26]	this work	1.57
NA/ethyl acetate	7.68 × 10^−4^	−2.66 × 10^−1^	288.15–318.15	this work	this work	17.5
CF/solvent						
SA/water	−7.30 × 10^−5^	−5.56 × 10^−3^	293.15–318.15	[26]	[26]	1.18
SA/acetonitrile	4.70 × 10^−5^	−2.38 × 10^−1^	293.15–318.15	[26]	[26]	2.65
SA/ethanol	2.63 × 10^−4^	−3.00 × 10^−1^	293.15–318.15	[26]	[26]	2.37
SA/ethyl acetate	1.53 × 10^−4^	−2.74 × 10^−1^	293.15–318.15	this work	this work	0.60
solvent/anti-solvent						
ethanol/water	0	−0.0382	343	[48]	[60]	
ethanol/acetonitrile	0	−0.005	293.15	[61]	[62]	
ethanol/ethyl acetate	0	−0.018	344.58–350.55	[61]	[62]	

^a^ Binary parameters should be used together with pure-component parameters from Table 2.

**Table 5:** List of symbols that refer to solubility points measured in a specific solvent or solvent/anti-solvent mixture with nicotinamide (NA), succinic acid (SA) or the cocrystal (CC) in the corresponding solid phases.

Symbol			Solvent or Solvent/Anti-Solvent Mixture
NA	SA	CC	
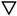	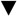	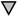	acetonitrile
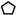	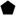	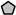	ethanol
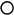	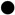	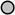	ethyl acetate
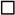	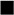	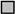	water
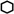	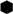	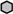	ethanol/ethyl acetate mixture 0.75/0.25 *w*/*w*
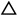	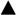	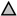	ethanol/ethyl acetate mixture 0.5/0.5 *w*/*w*
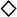	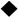	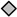	ethanol/ethyl acetate mixture 0.25/0.75 *w*/*w*
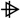	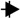	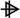	ethanol/acetonitrile 0.67/0.33 *w*/*w*
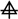	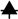	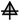	ethanol/water 0.5/0.5 *w*/*w*

**Table 6:** Average relative deviation (ARD) of calculated and experimental solubilities of nicotinamide (NA) and succinic acid (SA) in solvent/anti-solvent mixtures.

Component	Solvent/Anti-Solvent Mixture	Temperature (K)	ARD (%)
NA	ethanol/acetonitrile	298.15	0.04
NA	ethanol/ethyl acetate	298.15	4.24
NA	ethanol/ethyl acetate	310.15	5.86
NA	ethanol/water	298.15	5.42
SA	ethanol/acetonitrile	298.15	4.54
SA	ethanol/ethyl acetate	298.15	6.42
SA	ethanol/ethyl acetate	310.15	11.11
SA	ethanol/water	298.15	31.76

**Table 7:** Solubility product $K_{s}$ and reference enthalpy $\Delta h^{ref}$ of a cocrystal consisting of nicotinamide and succinic acid in a 2:1 stoichiometry used in this work.

$K_{s}$ (−)	Temperature (K)	$\Delta h^{ref}$ (kJ/mol)	Source for $\Delta h^{ref}$
3.70 × 10^−7^	298.15	64.75	[26]
1.00 × 10^−6^	310.15		

**Table 8:** Average relative deviation (ARD) of calculated and experimental solubilities of the nicotinamide/succinic acid 2:1 cocrystal (CC) in solvents and solvent/anti-solvent mixtures.

Component	Solvent or Solvent/Anti-Solvent Mixture	Temperature (K)	ARD (%)
CC	acetonitrile	298.15	5.26
CC	ethanol	298.15	1.28
CC	ethyl acetate	298.15	6.68
CC	water	298.15	2.58
CC	ethanol/acetonitrile 0.67/0.33 *w*/*w*	298.15	5.00
CC	ethanol/ethyl acetate 0.75/0.25 *w*/*w*	298.15	3.85
CC	ethanol/ethyl acetate 0.5/0.5 *w*/*w*	298.15	1.90
CC	ethanol/ethyl acetate 0.25/0.75 *w*/*w*	298.15	1.66
CC	ethanol/water 0.5/0.5 *w*/*w*	298.15	4.29
CC	ethanol	310.15	2.72
CC	ethyl acetate	310.15	1.81
CC	ethanol/ethyl acetate 0.5/0.5 *w*/*w*	310.15	7.31
CC	ethanol/ethyl acetate 0.25/0.75 *w*/*w*	310.15	2.51

## Supplementary Materials

Supplementary materials can be accessed at: http://www.mdpi.com/1420-3049/21/5/593/s1.

## Notation

The following notations are used in this manuscript:

a	molar Helmholtz energy (J·mol^−1^)
$a_{i}$	activity of component i (−)
$\Delta c_{P,i}^{SL}$	difference of the heat capacity of the solid and the liquid component i at its melting point (kJ·K^−1^·kg^−1^)
$\Delta h^{ref}$	reference enthalpy (kJ·kg^−1^)
$\Delta h_{i}^{SL}$	heat of fusion of component i (kJ·kg^−1^)
$k_{ij}\left(T\right)$	binary interaction parameter (−)
$k_{ij,slope}$	slope of the temperature-dependent binary interaction parameter (K^−1^)
$k_{ij,int}$	intercept of the temperature-dependent binary interaction parameter (K)
$K_{s}$	cocrystal solubility product (−)
$K_{s}^{\;ideal}$	cocrystal solubility product in an ideal solution (−)
$m_{i}^{seg}$	number of segments of component i (−)
$n_{exp}$	number of experimental data points (−)
R	gas constant (J·mol^−1^·K^−1^)
T	temperature (K)
$T_{i}^{SL}$	melting temperature of component i (K)
$T^{ref}$	reference temperature (K)
$\nu_{i}$	stoichiometric coefficient of component i (−)
$x_{i}$	mole fraction of component i (−)

## Abbreviations

The following abbreviations are used in this manuscript:

API	active pharmaceutical ingredient
CC	cocrystal
CF	coformer
NA	nicotinamide
SA	succinic acid
PC-SAFT	perturbed-chain statistical associating fluid theory
ARD	average relative deviation

## Greek Symbols

The following Greek symbols are used in this manuscript:

$\;\gamma_{i}$	activity coefficient of component *i* (−)
$u_{i}/k_{B}$	dispersion energy parameter (K)
$\varepsilon^{AiBi}/k_{B}$	association energy parameter (K)
$\kappa^{AiBi}$	association volume parameter (−)
σ	segment diameter (Å)

## Subscripts

The following subscripts are used in this manuscript:

i, j	component i, component j
