# Supplementary material for: Predicting the Solubility of Pharmaceutical Cocrystals in Solvent/Anti-Solvent Mixtures
Source: Molecules. 2016 May 7;21(5):593. doi: 10.3390/molecules21050593 (PMC6273375; doi:10.3390/molecules21050593)
Supplement: Supplementary file 1 [file molecules-21-00593-s001.pdf]

Supplementary Materials: Predicting the Solubility of Pharmaceutical Cocrystals in Solvent/Anti-Solvent Mixtures

Linda Lange, Stefan Heisel and Gabriele Sadowski

Table S1. Solubilities of nicotinamide in ethanol/ethyl acetate mixtures measured in this work in mole fractions (including standard deviation) at 298.15 K.

$w_{ethanol}$ in (Solute-Free) Solvent Mixture	$x^L_{nicotinamide}$
1	4.46×10^{-2} (3.08×10^{-6})
0.75	5.10×10^{-2} (2.48×10^{-5})
0.5	4.90×10^{-2} (1.88×10^{-5})
0.25	3.40×10^{-2} (1.69×10^{-5})
0	7.74×10^{-3} (2.17×10^{-5})

Table S2. Solubilities of nicotinamide in ethanol/ethyl acetate mixtures measured in this work in mole fractions (including standard deviation) at 310.15 K.

$w_{ethanol}$ in (Solute-Free) Solvent Mixture	$x^L_{nicotinamide}$
1	6.61×10^{-2} (2.80×10^{-5})
0.5	6.61×10^{-2} (2.70×10^{-5})
0.25	4.60×10^{-2} (8.61×10^{-6})
0	1.13×10^{-2} [1]

Table S3. Solubilities of nicotinamide in ethanol/water mixtures measured in this work in mole fractions (Including standard deviation) at **298**.15 K.

$w_{ethanol}$ in (Solute-Free) Solvent Mixture	$x^L_{nicotinamide}$
1	4.46×10^{-2} (3.08×10^{-6})
0.5	1.20×10^{-1} (9.81×10^{-4})
0	1.04×10^{-1} [1]

Table S4. Solubilities of nicotinamide in ethanol/acetonitrile mixtures measured in this work in mole fractions (including standard deviation) at 298.15 K.

$w_{ethanol}$ in (Solute-Free) Solvent Mixture	$x^L_{nicotinamide}$
1	4.46×10^{-2} (3.08×10^{-6})
0.66	5.61×10^{-2} (4.18×10^{-4})
0	8.65×10^{-3} (1.56×10^{-5})

Table S5. Solubilities of succinic acid in ethanol/ethyl acetate mixtures measured in this work in mole fractions (including standard deviation) at 298.15 K.

$w_{ethanol}$ in (Solute-Free) Solvent Mixture	$x^L_{succinic\ acid}$
1	4.20×10^{-2} (1.60×10^{-3})
0.75	4.69×10^{-2} (1.32×10^{-3})
0.5	4.60×10^{-2} (5.93×10^{-4})
0.25	3.10×10^{-2} (1.15×10^{-3})
0	3.87×10^{-3} (1.88×10^{-5})

Table S6. Solubilities of succinic acid in ethanol/ethyl acetate mixtures measured in this work in mole fractions (including standard deviation) at 310.15 K.

$w_{ethanol}$ in (Solute-Free) Solvent Mixture	$x^L_{succinic\ acid}$
1	5.73×10^{-2} (2.70×10^{-3})
0.5	6.01×10^{-2} (1.71×10^{-3})
0.25	4.75×10^{-2} (2.79×10^{-3})
0	5.46×10^{-3} (1.28×10^{-5})

Table S7. Solubilities of succinic acid in ethanol/water mixtures measured in this work in mole fractions (including standard deviation) at 298.15 K.

$w_{ethanol}$ in (Solute-Free) Solvent Mixture	$x^L_{succinic\ acid}$
1	4.20×10^{-2} (1.60×10^{-3})
0.5	4.10×10^{-2} (1.58×10^{-4})
0	1.67×10^{-2} (4.93×10^{-4})

Table S8. Solubilities of succinic acid in ethanol/acetonitrile mixtures measured in this work in mole fractions (including standard deviation) at 298.15 K.

$w_{ethanol}$ in (Solute-Free) Solvent Mixture	$x^L_{succinic\ acid}$
1	4.20×10^{-2} (1.60×10^{-3})
0.66	4.60×10^{-2} (3.81×10^{-4})
0	3.44×10^{-3} (1.72×10^{-5})

Table S9. Solubilities of the nicotinamide/succinic acid cocrystal system in ethanol measured in this work in mole fractions (including standard deviation) at 298.15 K.

$x^L_{succinic\ acid}$	$x^L_{nicotinamide}$	Solid Phase
2.68×10^{-2} (2.04×10^{-5})	3.84×10^{-3} (1.84×10^{-5})	cocrystal
2.02×10^{-2} (5.79×10^{-5})	4.15×10^{-3} (1.29×10^{-5})	cocrystal
7.59×10^{-3} (1.75×10^{-4})	7.31×10^{-3} (7.34×10^{-7})	cocrystal
1.73×10^{-3} (1.11×10^{-4})	1.44×10^{-2} (5.61×10^{-5})	cocrystal
8.86×10^{-4} (1.77×10^{-5})	1.89×10^{-2} (1.50×10^{-4})	cocrystal
4.20×10^{-2} (1.60×10^{-3})	0	succinic acid
0	4.46×10^{-2} (3.08×10^{-6})	nicotinamide

Table S10. Solubilities of the nicotinamide/succinic acid cocrystal system in ethanol/ethyl acetate (0.75/0.25 *w*/*w*) measured in this work in mole fractions (including standard deviation) at 298.15 K.

$x^L_{succinic\ acid}$	$x^L_{nicotinamide}$	Solid Phase
4.87×10^{-2} (3.42×10^{-4})	4.24×10^{-3} (2.75×10^{-5})	cocrystal
3.47×10^{-2} (7.21×10^{-5})	4.54×10^{-3} (2.01×10^{-5})	cocrystal
2.34×10^{-2} (1.42×10^{4})	5.11×10^{-3} (5.19×10^{-6})	cocrystal
1.01×10^{-2} (5.35×10^{-5})	6.57×10^{-3} (3.63×10^{-5})	cocrystal
4.51×10^{-3} (1.90×10^{-4})	9.91×10^{-3} (9.83×10^{-6})	cocrystal
1.54×10^{-3} (9.19×10^{-6})	1.76×10^{-2} (1.32×10^{-5})	cocrystal
7.21×10^{-4} (5.69×10^{-6})	3.21×10^{-2} (7.60×10^{-5})	cocrystal
4.29×10^{-4} (4.58×10^{-5})	4.49×10^{-2} (4.02×10^{-5})	cocrystal
4.69×10^{-2} (1.32×10^{-3})	0	succinic acid
0	5.10×10^{-2} (2.48×10^{-5})	nicotinamide

Table S11. Solubilities of the nicotinamide/succinic acid cocrystal system in ethanol/ethyl acetate (0.50/0.50 *w*/*w*) measured in this work in mole fractions (including standard deviation) at 298.15 K.

$x^L_{succinic\ acid}$	$x^L_{nicotinamide}$	**Solid Phase**
1.93×10^{-2} (4.71×10^{-4})	5.38×10^{-3} (4.23×10^{-5})	cocrystal
1.20×10^{-2} (2.09×10^{-4})	6.00×10^{-3} (1.12×10^{-5})	cocrystal
2.56×10^{-3} (1.05×10^{-4})	1.47×10^{-2} (8.47×10^{-5})	cocrystal
3.86×10^{-4} (2.01×10^{-4})	2.68×10^{-2} (1.19×10^{-5})	cocrystal
3.97×10^{-4} (2.33×10^{-5})	4.05×10^{-2} (1.36×10^{-4})	cocrystal
4.60×10^{-2} (5.93×10^{-4})	0	succinic acid
0	4.90×10^{-2} (1.88×10^{-5})	nicotinamide

Table S12. Solubilities of the nicotinamide/succinic acid cocrystal system in ethanol/ethyl acetate (0.25/0.75 *w*/*w*) measured in this work in mole fractions (including standard deviation) at 298.15 K.

$x^L_{succinic\ acid}$	$x^L_{nicotinamide}$	**Solid Phase**
6.11×10^{-3} (7.41×10^{-5})	5.24×10^{-3} (7.22×10^{-5})	cocrystal
2.72×10^{-3} (1.21×10^{-6})	7.75×10^{-3} (2.98×10^{-6})	cocrystal
9.27×10^{-4} (9.46×10^{-6})	1.44×10^{-2} (4.25×10^{-5})	cocrystal
4.78×10^{-4} (1.11×10^{-5})	2.17×10^{-2} (8.65×10^{-6})	cocrystal
3.19×10^{-4} (1.34×10^{-6})	2.94×10^{-2} (4.14×10^{-5})	cocrystal
3.10×10^{-2} (1.15×10^{-3})	0	succinic acid
0	3.40×10^{-2} (1.69×10^{-5})	nicotinamide

Table S13. Solubilities of the nicotinamide/succinic acid cocrystal system in ethyl acetate measured in this work in mole fractions (including standard deviation) at 298.15 K.

$x^L_{succinic\ acid}$	$x^L_{nicotinamide}$	**Solid Phase**
1.58×10^{-4} (1.10×10^{-5})	1.60×10^{-3} (8.66×10^{-6})	cocrystal
8.44×10^{-5} (2.48×10^{-5})	2.13×10^{-3} (2.59×10^{-6})	cocrystal
6.10×10^{-5} (6.10×10^{-5})	3.14×10^{-3} (9.90×10^{-6})	cocrystal
3.87×10^{-3} (1.88×10^{-5})	0	succinic acid
0	7.74×10^{-3} (2.17×10^{-5})	nicotinamide

Table S14. Solubilities of the nicotinamide/succinic acid cocrystal system in acetonitrile measured in this work in mole fractions (including standard deviation) at 298.15 K.

$x^L_{succinic\ acid}$	$x^L_{nicotinamide}$	**Solid Phase**
2.50×10^{-3} (3.74×10^{-5})	6.46×10^{-4} (9.23×10^{-7})	cocrystal
7.11×10^{-4} (3.56×10^{-5})	9.00×10^{-4} (1.38×10^{-6})	cocrystal
1.97×10^{-5}	4.68×10^{-3} (2.09×10^{-4})	cocrystal
3.44×10^{-3} (1.72×10^{-5})	0	succinic acid
0	8.65×10^{-3} (1.56×10^{-5})	nicotinamide

Table S15. Solubilities of the nicotinamide/succinic acid cocrystal system in ethanol/acetonitrile (0.66/0.34 *w*/*w*) measured in this work in mole fractions (including standard deviation) at 298.15 K.

$x^L_{succinic\ acid}$	$x^L_{nicotinamide}$	**Solid Phase**
3.41×10^{-2} (1.49×10^{-4})	4.51×10^{-3} (1.43×10^{-4})	cocrystal
1.88×10^{-2} (4.41×10^{-5})	5.81×10^{-3} (4.26×10^{-5})	cocrystal
8.21×10^{-3} (1.47×10^{-5})	8.61×10^{-3} (3.72×10^{-7})	cocrystal
2.84×10^{-3} (3.64×10^{-5})	1.46×10^{-2} (1.16×10^{-5})	cocrystal
1.02×10^{-3} (2.03×10^{-5})	2.39×10^{-2} (6.31×10^{-5})	cocrystal
8.01×10^{-4} (1.23×10^{-6})	3.53×10^{-2} (9.21×10^{-5})	cocrystal
4.60×10^{-2} (3.81×10^{-4})	0	succinic acid
0	5.61×10^{-2} (4.18×10^{-4})	nicotinamide

Table S16. Solubilities of the nicotinamide/succinic acid cocrystal system in ethanol/water (0.5/0.5 *w*/*w*) measured in this work in mole fractions (including standard deviation) at 298.15 K.

$x^L_{succinic\ acid}$	$x^L_{nicotinamide}$	**Solid Phase**
3.91×10^{-2} (2.25×10^{-3})	1.10×10^{-2} (1.71×10^{-5})	cocrystal
2.51×10^{-2} (9.57×10^{-5})	1.18×10^{-2} (1.45×10^{-5})	cocrystal
1.44×10^{-2} (7.08×10^{-5})	1.65×10^{-2} (1.05×10^{-5})	cocrystal
3.59×10^{-3} (5.76×10^{-5})	3.35×10^{-2} (2.97×10^{-4})	cocrystal
1.57×10^{-3} (4.39×10^{-6})	6.57×10^{-2} (6.65×10^{-5})	cocrystal
4.10×10^{-2} (1.58×10^{-4})	0	succinic acid
0	1.20×10^{-1} (9.81×10^{-4})	nicotinamide

Table S17. Solubilities of the nicotinamide/succinic acid cocrystal system in water measured in this work in mole fractions (including standard deviation) at 298.15 K.

$x^L_{succinic\ acid}$	$x^L_{nicotinamide}$	**Solid Phase**
3.62×10^{-4} (5.56×10^{-5})	4.37×10^{-2} (1.36×10^{-6})	cocrystal
1.00×10^{-2} (2.95×10^{-5})	3.48×10^{-3} (1.34×10^{-4})	cocrystal
5.92×10^{-4} (4.07×10^{-5})	3.56×10^{-2} (3.46×10^{-5})	cocrystal
5.38×10^{-3} (9.12×10^{-5})	4.65×10^{-3} (6.62×10^{-7})	cocrystal
1.67×10^{-2} (4.93×10^{-4})	0	succinic acid
0	1.04×10^{-1} [1]	nicotinamide

Table S18. Solubilities of the nicotinamide/succinic acid cocrystal system in ethanol measured in this work in mole fractions (including standard deviation) at 310.15 K.

$x^L_{succinic\ acid}$	$x^L_{nicotinamide}$	**Solid Phase**
4.74×10^{-2} (1.73×10^{-3})	6.01×10^{-3} (2.56×10^{-4})	cocrystal
2.38×10^{-2} (2.01×10^{-4})	7.24×10^{-3} (1.16×10^{-5})	cocrystal
4.46×10^{-3} (1.24×10^{-4})	1.35×10^{-2} (6.52×10^{-6})	cocrystal
5.66×10^{-4} (1.93×10^{-4})	4.42×10^{-2} (5.79×10^{-4})	cocrystal
5.73×10^{-2} (2.70×10^{-3})	0	succinic acid
0	6.61×10^{-2} (2.80×10^{-5})	nicotinamide

Table S19. Solubilities of the nicotinamide/succinic acid cocrystal system in ethanol/ethyl acetate (0.50/0.50 *w*/*w*) measured in this work in mole fractions (including standard deviation) at 310.15 K.

$x^L_{succinic\ acid}$	$x^L_{nicotinamide}$	**Solid Phase**
4.14×10^{-2} (3.17×10^{-4})	7.31×10^{-3} (6.33×10^{-6})	cocrystal
1.98×10^{-2} (1.02×10^{-4})	8.96×10^{-3} (1.14×10^{-5})	cocrystal
4.44×10^{-3} (8.27×10^{-5})	1.68×10^{-2} (6.43×10^{-5})	cocrystal
1.18×10^{-3} (2.62×10^{-5})	3.90×10^{-2} (8.82×10^{-5})	cocrystal
6.01×10^{-2} (1.71×10^{-3})	0	succinic acid
0	6.61×10^{-2} (2.70×10^{-5})	nicotinamide

Table S20. Solubilities of the nicotinamide/succinic acid cocrystal system in ethanol/ethyl acetate (0.25/0.75 *w*/*w*) measured in this work in mole fractions (including standard deviation) at 310.15 K.

$x^L_{succinic\ acid}$	$x^L_{nicotinamide}$	**Solid Phase**
3.70×10^{-2} (2.29×10^{-4})	4.04×10^{-3} (4.86×10^{-5})	cocrystal
1.74×10^{-2} (2.65×10^{-5})	4.60×10^{-3} (8.72×10^{-5})	cocrystal
2.62×10^{-3} (6.92×10^{-5})	1.01×10^{-2} (1.30×10^{-5})	cocrystal
3.25×10^{-4} (7.49×10^{-5})	3.34×10^{-2} (7.26×10^{-5})	cocrystal
4.75×10^{-2} (2.79×10^{-3})	0	succinic acid
0	4.60×10^{-2} (8.61×10^{-6})	nicotinamide

PXRD Pattern

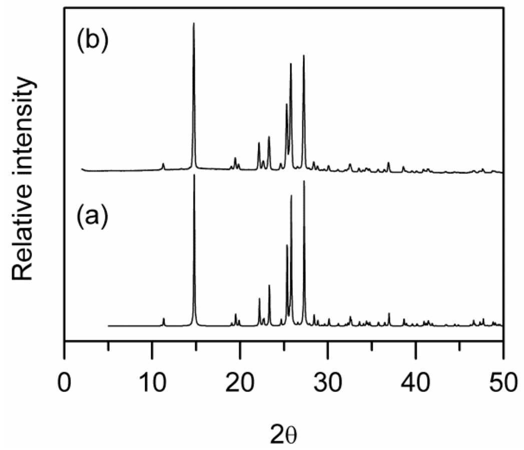

Figure S1. Representative PXRD patterns of nicotinamide (**a**) simulated from single-crystal data [2] and (**b**) experimental.

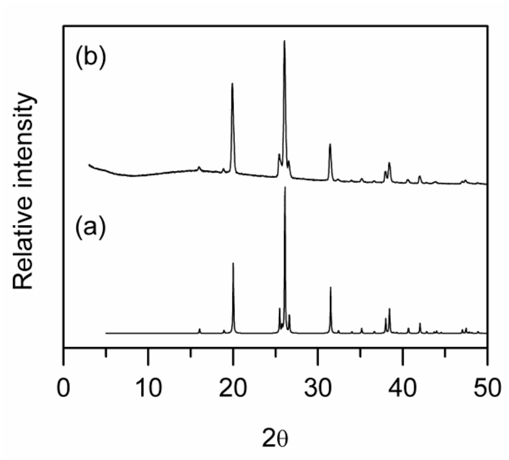

Figure S2. Representative PXRD patterns of succinic acid (**a**) simulated from single-crystal data [3] and (**b**) experimental.

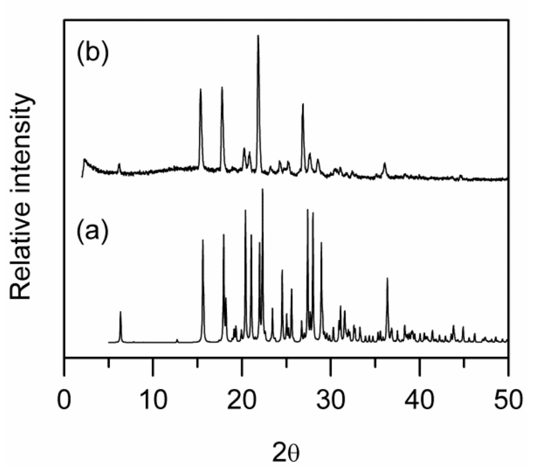

Figure S3. Representative PXRD patterns of cocrystal containing of nicotinamide and succinic acid (2:1) (**a**) simulated from single-crystal data [4] and (**b**) experimental.

DSC Pattern

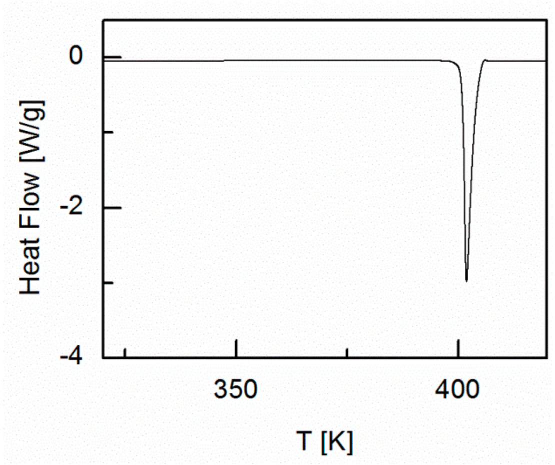

Figure S4. DSC data (heat flow) of nicotinamide.

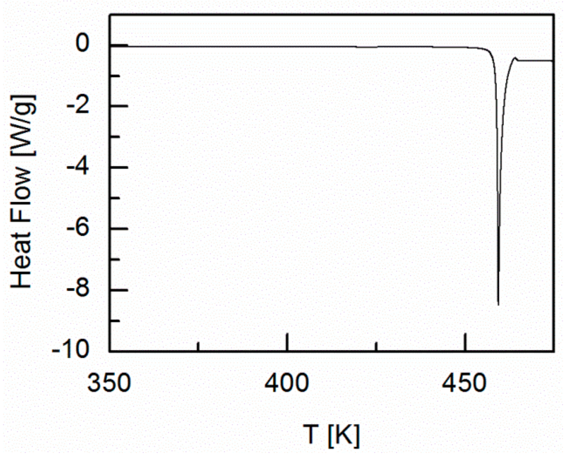

Figure S5. DSC data (heat flow) of succinic acid.

References

1. Klein, T.; Laube, F.; Prudic, A. *Laboratory of Thermodynamics*; TU Dortmund: Dortmund, Germany, 2013.
2. Miwa, Y.; Mizuno, T.; Tsuchida, K.; Taga, T.; Iwata, Y. Experimental charge density and electrostatic potential in nicotinamide. *Acta Cryst. C* **1999**, 55, 78–84.
3. Leviel, J.-L.; Auvert, G.; Savariault, J.-M. Hydrogen bond studies. A neutron diffraction study of the structures of succinic acid at 300 and 77 K. *Acta Cryst. B* **1981,** 37, 2185–2189.
4. Thompson, L. J.; Voguri, R. S.; Cowell, A.; Male, L.; Tremayne, M. The cocrystal nicotinamide–succinic acid (2/1). *Acta Cryst. C* **2010**, *66*, o421–o424.
